# Melanoma antigen A12 regulates cell cycle via tumor suppressor p21 expression

**DOI:** 10.18632/oncotarget.19497

**Published:** 2017-07-22

**Authors:** Teruki Yanagi, Ko Nagai, Hiroshi Shimizu, Shu-Ichi Matsuzawa

**Affiliations:** ^1^ Sanford Burnham Prebys Medical Discovery Institute, La Jolla, California, USA; ^2^ Department of Dermatology, Hokkaido University Graduate School of Medicine, Sapporo, Japan; ^3^ Department of Neurology, Kyoto University Graduate School of Medicine, Kyoto, Japan

**Keywords:** MAGEA12, cell cycle, p21, cyclin dependent kinase, ubiquitination

## Abstract

Melanoma-associated antigen family A (MAGE-A) is a family of cancer/testis antigens that are expressed in malignant tumors but not in normal tissues other than the testes. MAGE-A12 is a MAGE-A family gene whose tumorigenic function in cancer cells remains unclear. Searches of the Oncomine and NextBio databases revealed that malignant tumors show up-regulation of *MAGE-A12* mRNA relative to corresponding normal tissue. In PPC1 primary prostatic carcinoma cells and in HCT116 colorectal cancer cells (wild type and p53-depleted), *MAGE-A12* gene knockdown using siRNA or shRNA diminishes cancer cell proliferation as assessed by cellular ATP levels, cell counting, and clonogenic assays. FACS analyses of annexin V-PI staining and DNA content show that *MAGE-A12* knockdown causes G2/M arrest and apoptosis. In tumor xenografts of HCT116 cells, conditional knockdown of *MAGE-A12* suppresses tumor growth. The depletion of *MAGE-A12* leads to the accumulation of tumor suppressor p21 in PPC1, HCT116, and p53-depleted HCT116 cells. Conversely, *CDKN1A* knockdown partially rescues the viability of PPC1 cells transfected with siRNA targeting *MAGE-A12*, while p21 overexpression leads to proliferation arrest in PPC-1 cells. Furthermore, exogenous MAGE-A12 expression promotes the ubiquitination of p21. Our findings reveal that MAGE-A12 plays crucial roles in p21 stability and tumor growth, suggesting that MAGE-A12 could provide a novel target for cancer treatment.

## INTRODUCTION

The melanoma-associated antigen (MAGE) protein family is a large, highly conserved group of proteins that share a MAGE homology domain [1]. The human MAGE family proteins can be divided into two groups based on expression pattern. Type I MAGE proteins are cancer/testis antigens and include the MAGE-A, -B, and -C subfamilies, which are clustered on the X-chromosome. Type II MAGE proteins are expressed throughout tissues and are not limited to the X chromosome [1]. Members of melanoma-associated antigen family A (MAGE-A) are expressed in a wide variety of malignant tumors but not in normal adult tissues other than the testes [2]. Higher MAGE-A expression correlates with a poor outcome for patients with certain cancers, such cancers of the breast, prostate, and lungs [3–7]. The expression of MAGE-A family members is increased in cancer stem-like cell populations [8]. Recently, some MAGE-A proteins have been reported to regulate the ubiquitination of the p53, thereby regulating cancer cell proliferation [9–11]. However, most of the functions of MAGE-A proteins have not been investigated in detail. MAGE-A12, a MAGE-A family gene, has similar amino acid sequences to other MAGE-A family proteins, especially MAGE-A2, -A3, and A-6 [2]. MAGE-A12 is expressed in malignant tumors, but not in non-neoplastic normal tissues [12, 13]; however, its tumorigenic function remains unclear.

p21 (also known as Cip1, Waf1, cyclin-dependent kinase inhibitor 1A) is a tumor suppressor that regulates cell proliferation [14]. p21 binds to and inhibits the kinase activity of Cdk2 and Cdk1, leading to cell cycle arrest. Furthermore, p21 is associated with not only G2 arrest but also senescence [15]. In contrast to the conventional tumor suppressor p53, loss of p21 expression commonly occurs not through genetic mutation, but through transcriptional or post-transcriptional regulation. Much of the control of p21 expression is at the transcriptional level, especially p53-dependent regulation. p21 expression is also controlled via post-transcriptional ubiquitin-dependent or independent proteolysis; however, the details of the mechanisms have not been elucidated.

We here in report here investigations on the role of MAGE-A12 in tumorigenesis that provide evidence that MAGE-A12 plays a crucial role in the proliferation of some types of cancer cells. *MAGE-A12*-depleted cancer cells show G2/M phase arrest, followed by apoptosis. *MAGE-A12* knockdown leads to the accumulation of tumor suppressor p21 without mRNA up-regulation. Knockdown of *CDKN1A* rescues the cell viability of cancer cells transfected with siRNA targeting MAGE-A12. Moreover, CMV promoter driving p21 overexpression leads to proliferation arrest in PPC1 cells. In tumor xenografts, the conditional knockdown of *MAGE-A12* suppresses tumor growth. Taken together, these findings reveal an unexpected role for MAGE-A12 in cancer cell proliferation, suggesting that these molecules may provide novel targets for the future discovery of oncology therapies.

## RESULTS

### MAGE-A12 is overexpressed in malignant tumors and associated with poor patient-prognosis

To assess the role of MAGE-A12 in normal tissues and cancers, we examined a public database to evaluate the levels of *MAGE-A12* expression. The Oncomine database (https://www.oncomine.org/) showed MAGE-A12 to be expressed only in the testes and not in any other organs (Supplementary Figure 1), which is consistent with previous studies showing that MAGE-A family genes are expressed in only cancer or the testes. The NextBio database (https://www.nextbio.com/), a TCGA database containing information on *MAGE-A12* RNA expression levels, showed *MAGE-A12* to be an up-regulated gene in cancers, relative to corresponding normal tissue (Supplementary Figure 2A). The prognostic value of *MAGE-A12* was assessed using the Kaplan-Meier Plotter (http://kmplot.com/analysis/), an online tool to correlate survival with gene expression, based upon microarray data from 1,405 patients with lung cancer. High *MAGE-A12* mRNA levels were significantly correlated with lower overall survival in lung cancer (Supplementary Figure 2B). Similar results were observed in the patients with gastric cancer (Supplementary Figure 2C). This data from public databases indicates that MAGE-A12 is over-expressed in malignant tumors and is associated with poor patient-prognosis. Next, we assessed MAGE-A12 mRNA expression levels in various cell lines. Compared to normal cell lines (IMR-90 and 267B1 cells), two cancer cell lines (HCT116 and PPC1) showed higher MAGE-A12 expression levels (Supplementary Figure 3).

### Knockdown of MAGE-A12 regulates tumor cell growth and proliferation

**Figure 1:**
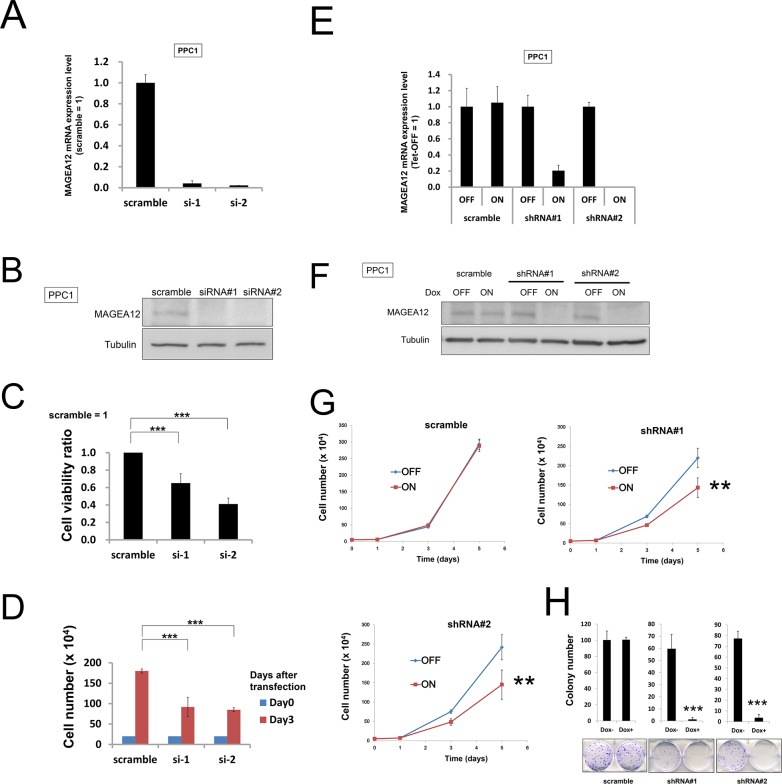
Knockdown of MAGE-A12 mitigates the growth of cancer cells **(A)** PPC1 cells transfected with scrambled RNA or two different siRNAs targeting MAGE-A12 (siRNAs#1, #2). After 48 hours, the relative levels of MAGE-A12 mRNA were measured by qRT-PCR analysis. **(B)** Cell lysates 48 hours after siRNA transfection were prepared and were normalized for total protein content, and aliquots were analyzed by immunoblotting using mouse anti-MAGE-A12 (top) or anti-beta-actin (bottom) antibodies. **(C)** PPC1 cells transfected with control RNA or various siRNAs targeting MAGE-A12. At 72 hours, cellular ATP levels were measured as a surrogate indicator of relative number of viable cells, with data expressed as the ratio of values for cells transfected with MAGE-A12 to values for the control siRNAs (mean ± SD; n = 3). *** p < 0.001 by t-test. **(D)** To measure cell growth, 2.0 × 10^5^ cells transfected with the indicated siRNAs were seeded onto 60-mm-diameter plates. At 72 hours, the numbers of cells were counted. *** p < 0.001 by t-test. **(E)** PPC1 cells stably containing inducible shRNAs targeting different sites on MAGE-A12 mRNA (shRNA#1, #2) were cultured for 48 hours with doxycycline (Dox: 100 ng/ml). Levels of MAGE-A12 mRNA were measured by qRT-PCR, with normalization relative to GADPH (mean ± SD; n = 2). **(F)** Protein lysates were generated from PPC1 cells (shRNA#1, #2, and scramble control) that had been cultured for 48 hours with or without 100 ng/ml doxycycline, normalized for total protein concentration, and analyzed by SDS-PAGE/immunoblotting using antibodies for MAGE-A12 (top) and beta-actin (bottom). **(G)** PPC1 cells (5.0 × 10^4^) stably containing inducible shRNAs were cultured in 60-mm-diameter plates for 24 hours and then stimulated with (ON) or without (OFF) 100 ng/ml doxycycline. The cells were counted at 1, 3, and 5 days after seeding (mean ± SD; n = 3). ** p < 0.01 by t-test. **(H)** PPC1 cells stably containing inducible shRNAs were seeded at 300 cells per well in 60-mm dishes. At 24 hours, doxycycline (ON: 100 ng/ml, OFF: 0 ng/ml) was added. Colonies consisting of > 50 cells were enumerated on day 10. All data represent mean ± SD (n = 3). *** p < 0.001 by t-test.

To investigate the effect of MAGE-A12 knockdown in cancer cells, we performed siRNA experiments using two siRNAs that target MAGE-A12. In human PPC1 primary prostatic cancer cells, quantitative RT-PCR and immunoblotting confirmed reduced levels of mRNA and protein by these siRNAs (Figure 1A and 1B). First, we analyzed cell viability changes in the presence of MAGE-A12 knockdown. Prostate cancer PPC1 cells were treated with negative control siRNAs or siRNAs targeting MAGE-A12, and cell viability was assessed 72 hours later. Cultures of MAGE-A12 knockdown PPC1 cells showed reduced relative numbers of viable cells compared to control cell cultures transfected with negative control siRNA, measured 3 days after transfection using ATP levels (Figure 1C). Similar results were obtained by direct cell counting methods, showing a reduction in the numbers of viable cells in cultures of PPC1 cells within 3 days of MAGE-A12 siRNA treatment (Figure 1D).

**Figure 2:**
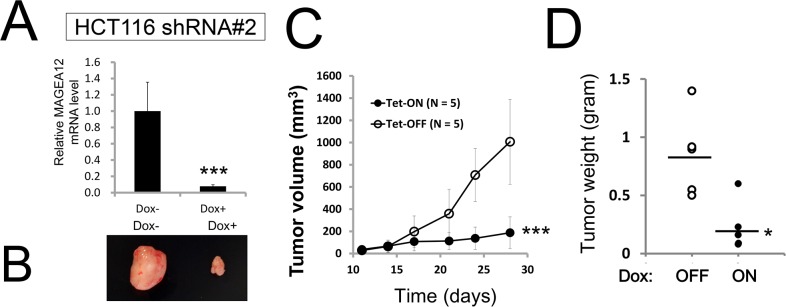
Knockdown of MAGE-A12 diminishes cancer cell growth *in vivo* **(A-D)** *In vivo* tumor xenograft analysis. HCT116 cells stably containing shRNA#2 (5.0 × 10^6^ cells resuspended in 200-μl serum-free RPMI) were subcutaneously injected into the flanks of 7-week-old female *nu/nu* mice. When tumor volumes reached 150-200 mm^3^, the animals were provided water with or without 2% doxycycline (“Dox”) (n = 5 mice per group). At 28 days, the relative levels of MAGE-A12 mRNA were measured by qRT-PCR analysis (A). A representative image of xenograft tumors (B). Tumor volumes (mm^3^) were measured every other day (mean ± SD; n = 5, C). *** p < 0.001 by t-test. (D) Tumors recovered from sacrificed mice at day 19 were weighed (mean = horizontal line). Differences between Dox-treated (+) versus Dox-untreated (−) groups were statistically significant (* p < 0.05, by t-test).

Next, to support the results of the siRNA experiments, we performed shRNA experiments in which the target sequences of shRNAs were different from those of the siRNAs. We used tetracycline-inducible shRNA vectors targeting MAGE-A12 to assess the impact of MAGE-A12 deficiency on the growth and survival of tumor cells. In PPC1 cells that were stably infected with a lentivirus expressing two different shRNAs targeting MAGE-A12, culturing with the tetracycline analog doxycycline resulted in reductions in MAGE-A12 mRNA levels (Figure 1E). Doxycycline-inducible reductions in MAGE-A12 protein were also observed (Figure 1F). In contrast, MAGE-A12-targeting shRNAs did not substantially reduce levels of mRNAs encoding MAGE-A2, -A3, or -A6 (Supplementary Figure 4). Compared to control cells (Tet-OFF), the growth rates of doxycycline-stimulated MAGE-A12 knockdown PPC-1 cells (Tet-ON) were significantly diminished at 5 days after seeding (Figure 1G). In clonogenic assays, the number of tumor cell colonies was significantly lower with MAGE-A12 knockdown (Tet-ON) than for control cells (Tet-OFF) (Figure 1H). Similar results were obtained using other tumor cell lines, such as human HCT116 colorectal cancer cells and isogenic p53-deficient cells (HCT116 p53-) (Supplementary Figures 5, 6). From these results, we conclude that MAGE-A12 regulates the proliferation and survival of cancer cells. To extend these studies into an *in vivo* context, we used HCT116 cells containing inducible MAGE-A12 shRNA (#2, doxycycline-induced knocked-down mRNA levels in shRNA#2 were as low as those in shRNA#1) in a tumor xenograft model. Immunocompromised null/null (*nu/nu*) mice were injected subcutaneously with HCT116 cells, and tumors were allowed to grow for 11 days before doxycycline was added to the drinking water for 17 days to induce the shRNA vector, which resulted in reduced MAGE-A12 mRNA expression in tumors (Figure 2A). Inducing MAGE-A12 shRNA expression remarkably suppressed tumor growth *in vivo* (Figure 2B-2D).

### Knockdown of MAGE-A12 results in G2/M arrest and apoptosis

**Figure 3:**
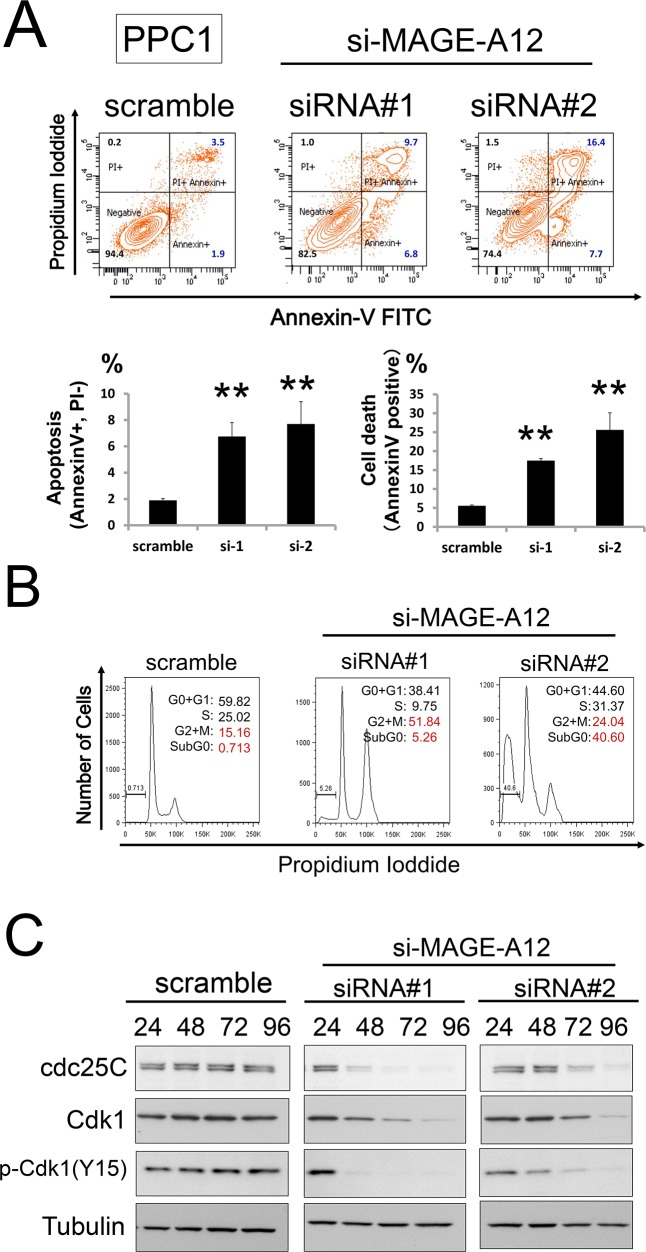
MAGEA12 regulates the cell cycle and apoptosis of cancer cells **(A, B)** Prostate cancer PPC1 cells were transfected with scrambled RNA or two different siRNAs targeting MAGE-A12 (siRNAs#1, #2). At 48 hours, FACS analysis was performed to assess the percentage of cells binding Annexin V. The % Annexin V-positive cells (A: apoptosis, B: cell death) were then assessed (mean ± SD; n = 3, ** p < 0.01). **(C)** PPC1 cells transfected with siRNAs were cultured for 48 hours, followed by FACS analysis of DNA content. Data represent relative DNA (propidium iodide fluorescence; x-axis) versus relative cell number (y-axis). **(D)** PPC1 cells transfected with control or siRNAs targeting MAGE-A12 were incubated for the indicated hours. Cell lysates were harvested, followed by immunoblotting with the indicated antibodies.

To examine the mechanisms of cell growth suppression by MAGE-A12 knockdown, a fluorescent annexin V/propidium iodide (PI) double-staining assay was performed, which showed increases in apoptosis over time in cultures of MAGE-A12 RNAi-treated PPC1 cells (Figure 3A). Cell cycle analyses were also performed using FACS-based DNA content analysis of PPC1 cells, which allowed the percentages of 2N, 4N, polyploid (DNA content > 4N), and hypoploid cells to be quantified (Figure 3B). In cultures of MAGE-A12 knockdown cells, the proportion of G2/M phase cells (4N DNA content) and the number of hypoploid (apoptotic) cells were increased at 72 hours. SDS-PAGE/immunoblot analyses using MAGE-A12 knockdown PPC1 cell lysate showed that cdc25C and phospho-Cdk1 (Y15) levels were down-regulated, which is consistent with FACS-based DNA content analysis (Figure 3C).

### MAGE-A12 knockdown leads to p21 accumulation

**Figure 4:**
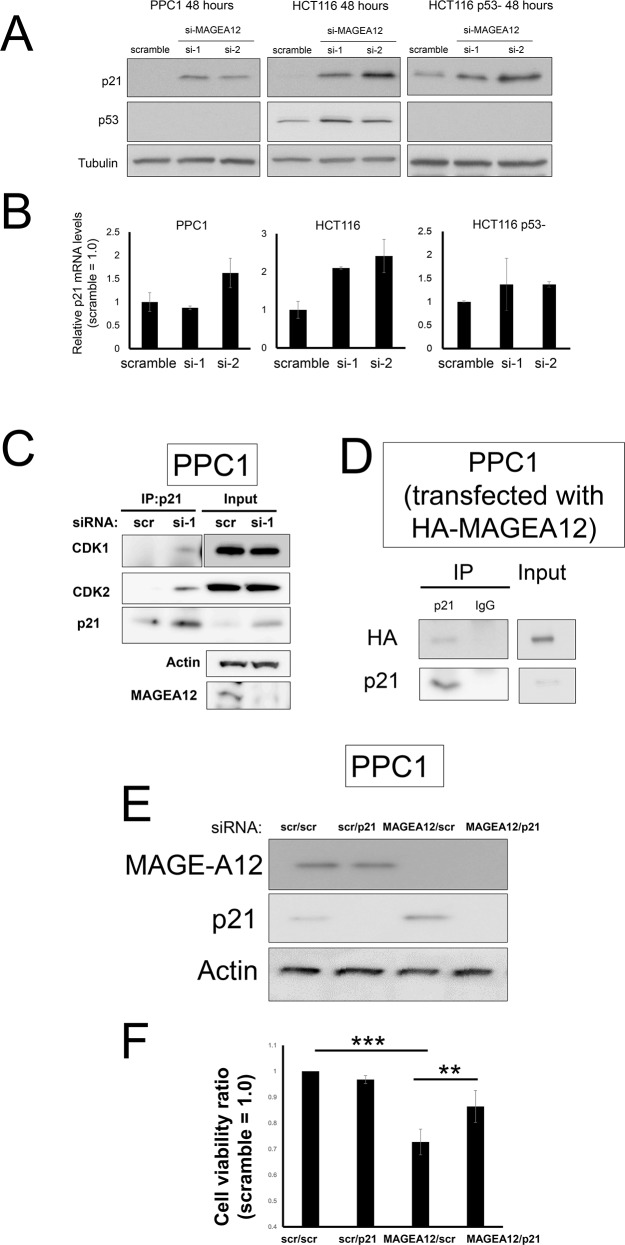
MAGE-A12 knockdown leads to accumulation of p21 **(A, B)** PPC1, HCT116, and isogenic p53-deficient HCT116 (HCT116 p53-) cells were transfected with control RNA or two different siRNAs targeting MAGE-A12. At 48 hours, cell lysates were prepared, normalized for total protein content, and analyzed by immunoblotting using antibodies for p21 (top), p53 (middle), and tubulin (bottom) (A). Also, mRNAs were extracted and levels of p21 mRNA were measured by qRT-PCR, with normalization relative to GADPH (mean ± SD; n = 2) (B). **(C)** PPC1 cells were reverse-transfected with control or siRNA targeting MAGE-A12 (si-1). At 48 hours, the cells were lysed in buffer containing 1% NP-40, and the cell lysates were subjected to immunoprecipitation (IP) with antibodies to p21 and the resulting precipitates were subjected to immunoblot analysis with antibodies to the indicated proteins. A portion (5%) of the lysates (“input”) was also subjected directly to immunoblot analysis with the same antibodies. **(D)** PPC1 cells were transfected with the pcDNA3-HA-MAGE-A12 vector. At 48 hours, the cells were lysed in buffer containing 1% NP-40, and the cell lysates were subjected to immunoprecipitation (IP) with antibodies to p21 or control rabbit IgG and the resulting precipitates were subjected to immunoblot analysis with anti-HA antibody. A portion (5%) of the lysates (“input”) was also subjected directly to immunoblot analysis. **(E, F)** PPC1 cells transfected with scramble-control RNA, or siRNAs targeting MAGE-A12 and p21 in various combinations as indicated (5 nM each, total siRNA concentration of 10 nM). At 72 hours, cell lysates were prepared, normalized for total protein content, and analyzed by immunoblotting using antibodies for MAGE-A12 (top), p21 (middle), and beta-actin (bottom) (E). (F) Cellular ATP levels were measured, with data expressed as the ratio of values for cells transfected with various siRNAs to values for the control siRNAs (mean ± SD; n = 3). ** p < 0.01, *** p < 0.001 by t-test.

To explore the mechanism by which knockdown of MAGE-A12 induces G2/M cell cycle arrest and apoptosis, we examined the impact of MAGE-A12 knockdown on the expression of various proteins that have been implicated in apoptosis and the cell cycle. siRNA or shRNA-mediated MAGE-A12 knockdown up-regulated expression levels of tumor suppressor p21 in PPC1, HCT116 cells, and HCT116 p53- cells (Figure 4A), while p21 mRNA levels were not notably up-regulated (Figure 4B). p21 accumulation was also observed in the shRNA-mediated MAGE-A12 knockdown cells (Supplementary Figure 7). In the HCT116 cells, MAGE-A12 knockdown also led to the up-regulation of p53, which is consistent with the function of other MAGE-A family proteins [9]. These results suggest that the elevated levels of p21 protein expression by MAGE-A12 knockdown have both a p53-dependent pathway and a p53-independent pathway. Next, we assessed the effect of MAGE-A12 on p21's interaction with Cdks, which p21 is known to bind and suppress. The p21 protein that accumulated in MAGE-A12 knockdown cells was found to interact with Cdk1 and Cdk2 (Figure 4C), suggesting that p21 retains its ability to suppress target Cdks. Interaction of MAGE-A12 with p21 was confirmed by a co-immunoprecipitation analysis using HA-tagged MAGE-A12 and endogenous p21 (Figure 4D). To confirm the functional role of p21 in proliferation and cell cycle arrest caused by MAGE-A12 knockdown, PPC1 cells were transfected with siRNAs targeting MAGE-A12, p21, or both, as well as with various control synthetic RNAs. Immunoblot analysis of the transfected cells confirmed MAGE-A12 and p21-knockdown after transfection (Figure 4E). In a cell viability assay, p21 knockdown restored cell viability in MAGE-A12 knockdown PPC1 cells (Figure 4F). Moreover, pcDNA3-vector-mediated p21 overexpression led to proliferation arrest and apoptosis in PPC1 cells (Supplementary Figure 8).

### MAGEA12 regulates p21 ubiquitination

p21 levels are regulated by ubiquitination/degradation mechanisms as well as by transcriptional regulation (Supplementary Figure 9A) [16]. The accumulation of p21 without the up-regulation of p21 mRNA strongly suggests that MAGE-A12 is involved in the process of p21 ubiquitination/degradation. MAGE-A family proteins have been reported to affect the ubiquitination of several proteins [9]. Thus, the effect of MAGE-A12 on p21 protein stability was examined with cyclohexamide (CHX) chase experiments. PPC1 and HCT116 cells transfected with siRNA targeting MAGE-A12 or a scramble-control were cultured for 48 hours, whereupon the cells were treated with 25 μg/ml cyclohexamide and the rate of p21 turnover was monitored (Supplementary Figure 9B). Endogenous p21 protein levels were significantly more stable in MAGE-A12 knockdown PPC1 cells than in control PPC1 cells, demonstrating that MAGE-A12 down-regulates p21 in a post-translational manner and showing that MAGE-A12 knockdown stabilizes the p21 protein. To examine whether MAGE-A12 is associated with the ubiquitination of p21, HEK293T cells were co-transfected with HA-ubiquitin, Myc-p21, and FLAG-MAGE-A12, followed by immunoprecipitation using anti-Myc antibody and by SDS-PAGE/immunoblotting analysis of the specimens. The ubiquitination of p21 was elevated in MAGE-A12-overexpressing cells (Supplementary Figure 9C). The ubiquitination of p21 appears to be mono-ubiquitination, which was recently reported to be associated with protein degradation [17].

## DISCUSSION

**Figure 5:**
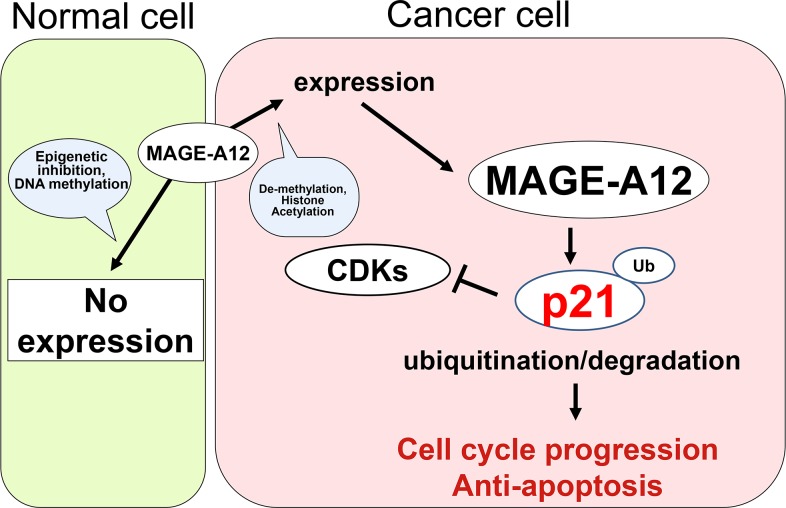
Model of the interaction between MAGE-A12 and p21 in cancer cells MAGE-A12 does not express in normal cells other than in the testes because of epigenetic inhibition (DNA methylation). However, in cancer cells, MAGE-A12 is expressed due to de-methylation or histone acetylation. MAGE-A12 plays a crucial role in the ubiquitination/degradation of tumor suppressor p21, which leads to cell-cycle progression and anti-apoptosis.

The present study showed that MAGE-A12 is expressed in only certain cancer cells and that it promotes the ubiquitination/degradation of tumor suppressor p21 (Figure 5). It has been known that Type I MAGE genes are located on the X chromosome and are expressed in the testes and placenta. The expression of Type I MAGE genes is silenced by DNA methylation; however, in malignant tumors, aberrant expression is observed. To date, the main focus on MAGE proteins has been on their potential for an anti-tumor vaccine. Concerning the physiological functions of MAGE family proteins, several studies show clues. Genome-wide yeast two-hybrid studies identified interactions between MAGE proteins and really interesting new gene (RING) proteins [18]. In 2010, MAGE proteins reported to interact with RING proteins and enhance E3 ligase mediated ubiquitination [9]. Moreover, in breast cancers, MAGE-A2, which is up-regulated in tamoxifen-resistant tumors, localizes to the nucleus and forms complexes with p53 and estrogen receptor alpha, resulting in repression of the p53 pathway [11]. Recently, MAGE-C2 has been reported to bind Rbx1 and regulate the cyclin E expression [19]. However, there are no detailed reports on the functions of MAGE-A12. In this report, we identified p21 as a target protein for the MAGE-A12-related ubiquitination pathway and showed that p21 levels are regulated by MAGE-A12 post-transcriptionally.

Our results also indicated that the accumulation of endogenous p21 induced by MAGE-A12 knockdown leads to G2/M arrest in tumor cells. Recently, Kreis et al. reviewed the role of p21 in the cell cycle and cell division in detail [20]. p21 is required in order to maintain G2 arrest by blocking the interaction of Cdk1 with Cdk activating kinase [21], which suggests that p21 accumulation is sufficient to arrest the cell cycle. That study is consistent with the present results, in which p21 that accumulates as a result of MAGE-A12 knockdown binds to Cdk1/Cdk2. Although the function of p21 during the G2/M phases has not been fully elucidated, our study suggests that MAGE-A12 is associated with p21 degradation at the G2/M phases.

Clinically, much effort has been directed towards MAGE-targeted immune therapies, but without success. In clinical trials for a lung cancer treatment, recombinant MAGE-A3 protein did not afford any greater disease-free survival than a placebo [22]. What is more, unanticipated death has been reported in T-cell-based anti-MAGE therapies [23, 24]. These large clinical studies revealed that immunotherapies using MAGE family proteins have some risks; thus, other approaches targeting MAGE family members directly should be developed. The aberrant expression of MAGE family proteins is common in various cancers; thus, MAGEA-targeted therapeutics will be applied to a wide range of cancer types. However, to date, no drugs have been reported to inhibit the interaction between MAGE-A family proteins and E3 ligases. Our study and previous studies suggest that MAGE-A proteins function as scaffold proteins for the degradation machinery of tumor suppressors (p53, p21). Therefore, the drug discovery of inhibitors targeting the interactions between MAGE proteins and their binding proteins could be a novel strategy for cancer therapies. To achieve such therapeutics, we need more investigations on the mechanisms of MAGE family proteins, including the protein binding sites and protein modifications. Furthermore, *in vivo* siRNA therapies targeting cancer cells have recently been developed [25]. Since the oncogenic functions of MAGE family proteins have been unmasked, such genes could be potential targets for siRNA therapies.

## METHODS

### Reagents and antibodies

The Cell Titer Glo cell viability assay kit was purchased from Promega. RNAiMAX and Lipofectoamine 2000 were obtained from Life Technologies. Opti-MEM was purchased from GIBCO. RPMI and DMEM media were purchased from Cellgro. Pre-designed small interfering RNA (siRNA) directed against human MAGE-A12 (silencer select, siRNA#1 (s8451): 5’-CACUCUAUUCUGUAAAUUU-3’, siRNA#2 (s8453): 5’-GGAGACGAGCUUCCAAGUA-3’), CDKN1A (p21, siRNA#1: s417), and a negative scramble control (#1) were purchased from Life Technologies. MG132 was purchased from Calbiochem. Antibodies against MAGE-A12 (mouse monoclonal, ab87973, Abcam), p53 (Ab-6, Merck), Cdk1 (610037, BD), Cdk2 (610145, BD), phospho-cdc2 (Y15) (#9111, CST), cdc25C (#4688, CST), alpha-tubulin (T5168, Sigma), FLAG (M2, Sigma), HA (3F10, Roche), Myc (9E10, Roche), beta-actin (Sigma), horseradish-peroxidase (HRP)-conjugated secondary antibodies (GE Health Care), and Alexa Fluor 488/594-conjugated secondary antibodies (Life Technologies) were purchased from the indicated sources.

### Cell lines and cell culture

PPC1, HCT116, and HCT116 p53−/− were cultured in RPMI or DMEM supplemented with 10% FBS (Sigma) and maintained at 37°C in a humidified atmosphere of 5% CO_2_ and 95% air.

### Plasmids

Full-length cDNAs encoding human MAGE-A12, CDKN1A (p21), and ubiquitin were purchased from Open Biosystems. Expression plasmids for various proteins were constructed in the pcDNA3 vector for transfection. Proper plasmid construction was confirmed by restriction enzyme digestion and DNA sequencing. DNA transfection was performed using Lipifectoamine2000 according to the manufacturer's instruction (Life Technologies).

### Cell viability assays using ATP measurement

Cell Titer Glo (Promega) was used for cell viability estimation. Cells were plated at a density of 5,000∼10,000 cells per well in 100 μL and cultured for 48 or 72 hours with or without treatment. The plates were then removed from the incubator and allowed to equilibrate to room temperature for about 10 minutes. Cell Titer Glo solution was added at 100 μl per well, and the plates were kept in the dark for 15 minutes before the luminescence was read by luminometer (Luminoskan Ascent; Thermo Scientific Corporation).

### Analysis of apoptosis

Cells were processed using the Annexin V-PI apoptosis assay kit (FITC-annexin V/propidium iodide staining) according to the manufacturer's protocol (Life Technologies) and 20,000 events were analyzed by flow cytometry using FACS Aria (Becton Dickinson).

### RNA interference

For transient knockdown, cells were transfected with siRNA duplexes by a reverse transfection method using Lipofectoamine RNAiMAX according to the manufacturer's instructions (Life Technologies). For each gene, up to 2 different siRNAs sequences were used. To confirm the siRNA silencing of genes, total protein or RNA was isolated 48 or 72 hours after transfection and was analyzed by immunoblotting or quantitative RT-PCR, respectively. The detailed protocol is provided below.

### Tet-inducible short hairpin RNA constructs, lentivirus and infection

A scramble-control (CAACAAGATGAAGAGCACCAA), MAGE-A12 shRNA#1 (GAGTGTGTTGGAGGCATCTGA), and MAGE-A12 shRNA#2 (AGACGAGCTTCCAAGTAGCAC) were cloned into inducible pLKO-Tet-On puromycin vectors as previously described [26, 27]. Lentiviral supernatants were generated according to an established protocol. Cells were selected by 1 μg/ml puromycin (MP Biomedicals) and expanded. shRNA was induced by the addition of 100 ng/ml doxycycline (Sigma) to the medium.

### Extraction of total RNA and quantitative RT-PCR analysis

We isolated total RNA from cultured cells and xenograft tumors using the RNeasy Plus Mini Kit (Qiagen). RNA concentrations were measured spectrophotometrically and samples were stored at −80°C until being used for RT-PCR. We reverse-transcribed RNA by using Superscript III (Life Technologies) according to the manufacturer's instructions. Complementary DNA samples were analyzed by the SYBR green system (Promega). The sequences for primers specific for human MAGE-A2, MAGE-A3, MAGE-A6, MAGE-A12, human CDKN1A (p21), and the control housekeeping genes for human GAPDH are as follows:

Human GAPDH

Forward: 5′- GAAGGTGAAGGTCGGAGTC -3′

Reverse: 5′- ATGGGATTTCCATTGATGAC -3′

Human MAGE-A2

Forward: 5′- GGTCGACAGATGCAGTGGT -3′

Reverse: 5′- CTGTCCCCCTCAGAACCTC -3′

Human MAGE-A3

Forward: 5′- GTGAGGAGGCAAGGTTCTGA -3′

Reverse: 5′- GGGCAATGGAGACCCACT -3′

Human MAGE-A6

Forward: 5′- GCCCTCTCACTTCCTCCTTC -3′

Reverse: 5′- GAGCTGGGCAATGGAGAC -3′

Human MAGE-A12

Forward: 5′- GTGGTCCTAAGATCTACCAAGCA -3′

Reverse: 5′- AGGGCAGCAGGTAGGAGTG -3′

Human CDKN1A (p21)

Forward: 5′- CCGAAGTCAGTTCCTTGTGG -3′

Reverse: 5′- CATGGGTTCTGACGGACAT -3′

All experiments were performed in either duplicate or triplicate and were normalized with respect to GAPDH levels.

### Laemmli SDS-PAGE, immunoblotting and immunoprecipitation

Cells were washed twice with PBS and harvested with radioimmunoprecipitation assay (RIPA) buffer composed of 20 mM Tris-HCl, pH 7.5, 150 mM NaCl, 0.1 mM EDTA, 1% Nonidet P-40, 0.1% SDS, 5 mM NaF and an EDTA-free complete protease cocktail tablet (Roche). The cells were left on ice for 20 min and centrifuged at 14,000 x *g* for 10 minutes. Protein concentrations were measured using the Bio-Rad protein assay kit (Bio-Rad). For Laemmli SDS-PAGE, proteins were separated on SDS-PAGE 4-15% gradient gels (Life Technologies) and transferred onto nitrocellulose membranes (Bio-Rad). Proteins were transferred to nitrocellulose membranes, which were blocked for 1 hour in Tris-buffered saline (TBS) with 0.05% Tween-20 and 5% non-fat dry milk, and were then incubated overnight at 4°C with primary antibodies diluted in blocking buffer. Membranes were rinsed three times in TBS with 0.05% Tween-20 and incubated with secondary HRP-conjugated antibodies for 1 hour at room temperature. An enhanced chemiluminescence (ECL) method (GE Health Care) was used for detection.

For immunoprecipitation (IP), cells were lysed in 1% NP-40 lysis buffer (20 mM Tris-HCl, pH 7.5, 150 mM NaCl, 0.1mM EDTA, 1% Nonidet P-40, 5 mM NaF and an EDTA-free cOmplete protease cocktail tablet). For analysis of ubiquitination of p21, cells were treated with 10 μM MG132 for 4 hours and lysed in RIPA buffer with 10 μM MG132. Three milligrams of protein lysate was used for immunoprecipitation by incubation with 2 μg of the antibody for 2 hours at 4°C. Then, 30 μl of protein G resin (Life Technologies) was added for 1 hour at 4°C and the IPs were washed four times with lysis buffer. Sample buffer was then added, and the beads were boiled for 10 minutes at 100°C. Samples were then analyzed by SDS-PAGE followed by immunoblotting.

### Clonogenic assay

Cells with Tet-inducible shRNA targeting MAGE-A12 were seeded at 500 cells per well in 6-well (35 mm) dishes. The cells were cultured with (ON) or without (OFF) doxycycline (100 ng/ml) for 14 days before being fixed and stained. For fixation, the cells were washed twice with PBS (pH 7.4) and were incubated with methanol at −20 °C for 20 minutes. Then, the cells were washed with PBS and incubated with 0.5% crystal violet dye in 25% methanol for 15 minutes. Finally, the dishes were immersed in tap water to remove excess crystal violet. A colony was defined as consisting of at least 50 cells.

### Cell growth assay

To measure cell growth rates, 1.0 × 10^5^ cells with Tet-inducible shRNA targeting MAGE-A12 were plated onto 60-mm-diameter plates with complete media with 10% FBS. After 24 hours, the culture media was changed to that with (ON) or without (OFF) doxycycline (100 ng/ml). The numbers of cells were counted at 1, 3, 5, and/or 7 days after seeding using the Countess automated cell counter (Life Technologies). In siRNA experiments, 1.0 × 10^5^ cells were reverse-transfected with siRNAs targeting MAGE-A12 or the scramble control. At 72 hours, the number of cells was counted.

### Cell cycle analysis by FACS

Cells were trypsinized, washed twice with PBS, fixed with cold 70% ethanol, and suspended in 100 μl of PBS with propidium iodide (20 μg/ml) and ribonuclease A (10 μg/ml). Then they were subjected to cell cycle analysis using FACS Aria (Becton Dickinson) and ModFitLT Software. A total 10,000 events were analyzed.

### Tumor xenograft experiments

All animal experiments were approved by the Institutional Animal Care and Use Committee of the Sanford-Burnham-Prebys Medical Discovery Institute. HCT116 cells (5.0 × 10^6^) with Tet-inducible shRNA targeting MAGE-A12 (shRNA#2) resuspended in 200-μl serum-free DMEM were injected subcutaneously into the flanks of 7-week-old female *nu/nu* mice using a 25-gauge needle. When the tumor volumes reached 150-200 mm^3^, the animals were given water with 2% doxycycline. Tumor size was measured every three days using calipers, and the tumor volume was calculated using the following formula: (long axis x short axis^2^)/2. The endpoint was set as tumor growth exceeding 1000 mm^3^ (Day 27 in the HCT116 xenograft).

### Statistical analysis

Statistical analysis was performed using the Excel add-in software Statcel® (OMS Ltd., Tokyo, Japan). Means and standard deviation (SD) were calculated statistically from three determinations. The data are expressed as mean ± SD. The chi-square goodness-of-fit test was used to determine whether the data were normally distributed. In this study, all the data were normally distributed; thus, we used t-tests (Student's or Welch's t) to assess the statistical significance of differences between various samples. p < 0.05 was considered significant.

### Gene expression

Individual cancer data sets were downloaded from Oncomine (https://www.oncomine.org), as previously described [26, 28]. The microarray dataset of MAGE-A12 mRNA expression in normal human organs was accessed using the Oncomine database [29] and was downloaded from NEBI-GEO (series GSE7307, measured by Human Genome U133 Plus 2.0 Array). Regarding box plots, the heavy line within the box indicates the median expression value for each organ. The upper and lower edges of the box show the 75^th^ and 25^th^ percentiles of the distribution, and the ends of the whiskers from the box indicate the 90^th^ and 10^th^ percentiles, respectively. The black dots represent maximum and minimum values. A student's t-test gave a P-value for the comparison of MAGE-A12 expression between testis and the others.

Publicly available datasets were identified using web-based NextBio Software (http://www.nextbio.com, [30]). The bar graph was derived from a previous study (study ID: TCGA – aggregate samples of 14 cancer RNA-seq expression profiles). The MAGE-A12 expression data were measured by Illumina Genome Analyzer RNA Sequencing Version 2 analysis and were obtained from patients in The Cancer Genome Atlas (TCGA). The datasets included samples for bladder cancer tumors (n = 223), breast cancer tumors (n = 1018), head and neck cancer tumors (n = 427), chromophobe kidney tumors (n = 66), kidney renal clear cell carcinomas (n = 519), kidney renal papillary cell carcinomas (n = 172), liver hepatocellular carcinomas (n = 482), prostate adenocarcinomas (n = 259), stomach adenocarcinomas (n = 285), and thyroid carcinomas (n = 500).

The prognostic significance of *MAGE-A12* in the overall survival of lung or gastric cancers was assessed using the Kaplan-Meier Plotter (http://kmplot.com/analysis/), which employs gene expression microarray data and survival information of patients with lung or gastric cancers downloaded from GEO (Affimetrix HGU133A, HGU 133+2, and HGU 133A 2.0) according to our previous report [26, 31].

## SUPPLEMENTARY MATERIALS FIGURES


