# Supplementary material for: Melanoma antigen A12 regulates cell cycle via tumor suppressor p21 expression
Source: Oncotarget. 2017 Jul 22;8(40):68448–59. doi: 10.18632/oncotarget.19497 (PMC5620269; doi:10.18632/oncotarget.19497)
Supplement: Supplementary file 1 [file oncotarget-08-68448-s001.pdf]

Melanoma antigen A12 regulates cell cycle via tumor suppressor p21 expression

SUPPLEMENTARY MATERIALS

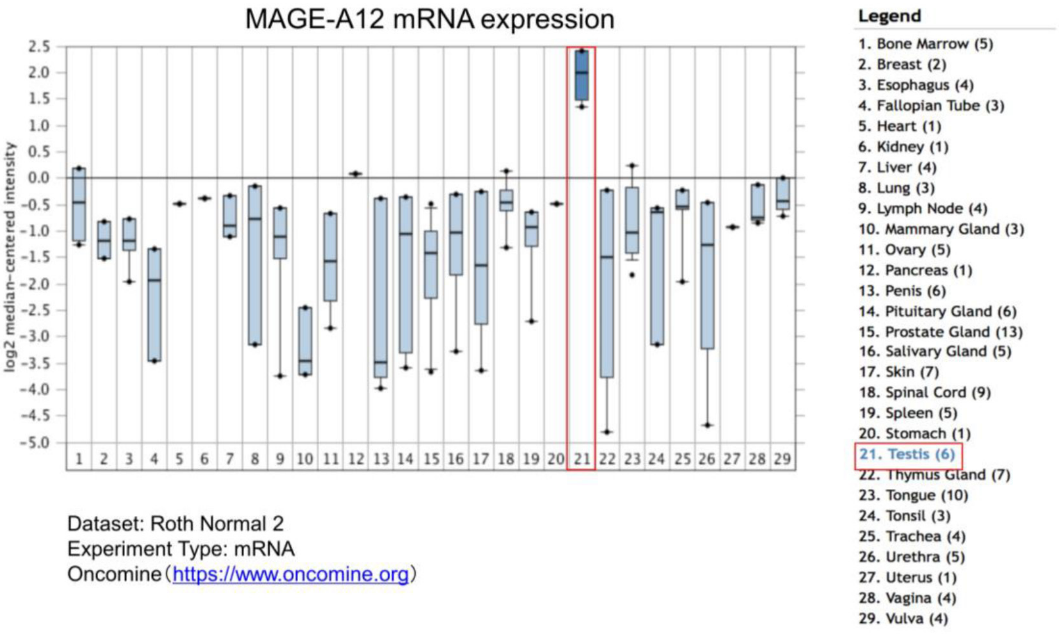

Supplementary Figure 1: MAGE-A12 is expressed in the testes. MAGE-A12 mRNA expression in each normal human organ is shown as box plots (as log2 median-centered intensity). The graph indicates that MAGE-A12 is expressed in the testes specifically (testes vs. other; P-value <0.001). The number in brackets after the sample name indicates the test sample number. The dataset was accessed using Oncomine (http://www.oncomine.org).

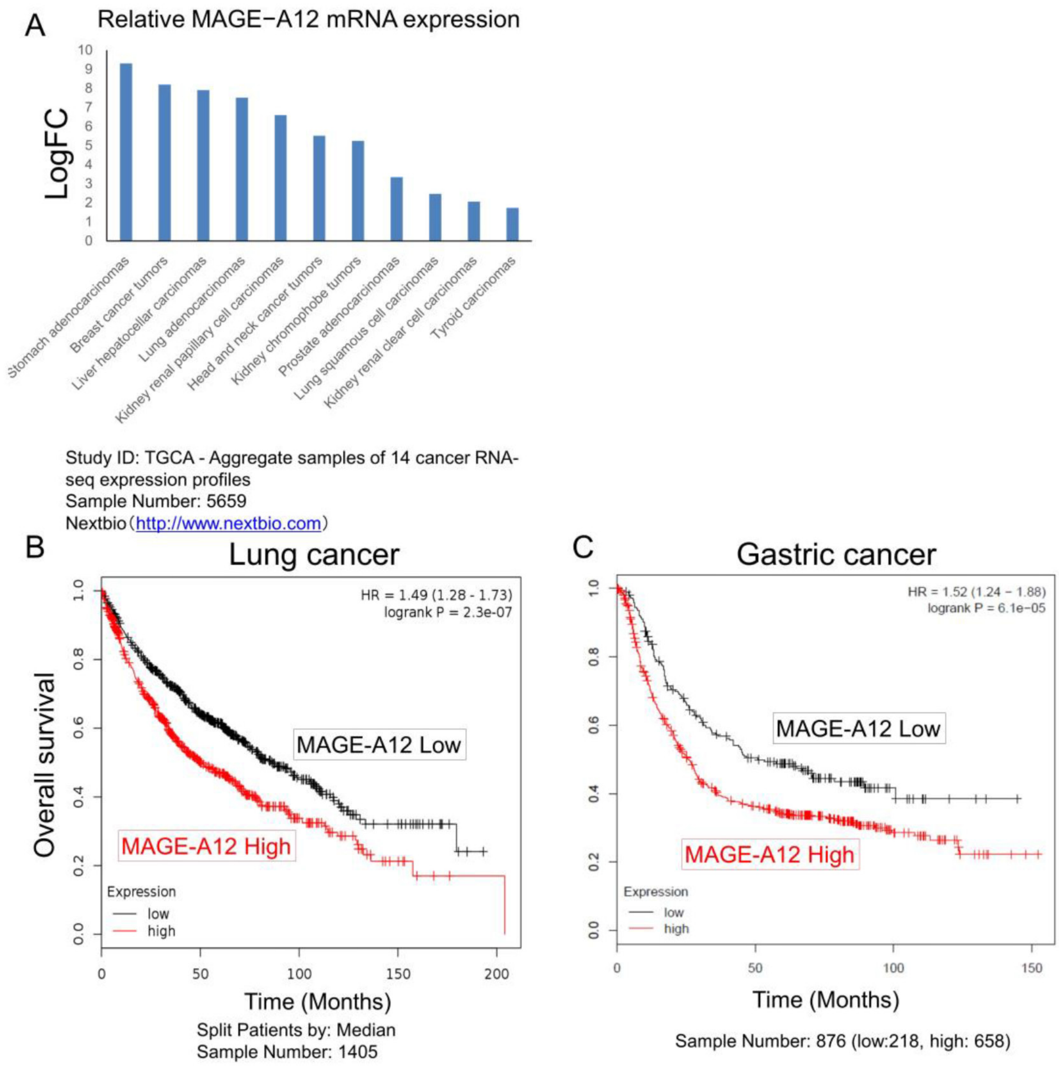

Supplementary Figure 2: MAGE-A12 expresses in cancer cells and correlates with poor prognosis. (A) MAGE-A12 mRNA expression in several cancer types was analyzed using NextBio software (https://www.nextbio.com). Each bar indicates a comparison between cancer tissue samples and corresponding normal tissue: For example, LogFC = 3 means MAGE-A12 RNA expression in cancer patients is 8 times that of normal. Each P-value (versus normal tissue), is less than 0.005. The datasets were obtained from patients in The Cancer Genome Atlas (TCGA). **(B, C)** High MAGE-A12 levels correlate with poor patient prognosis. Kaplan-Meier survival plots were obtained using Kaplan-Meier Plotter (http://www.kmplot.com) to display the probability of overall survival of 1,405 patients with lung cancer grouped according to MAGE-A12 dichotomized into high versus low expression median mRNA expression levels (B). Similar results were obtained in the overall survival of 876 patients with gastric cancer (C). Hazard ratio (HR) with 95% confidence intervals and P value for the comparison of the low and high MAGE-A12 groups are shown.

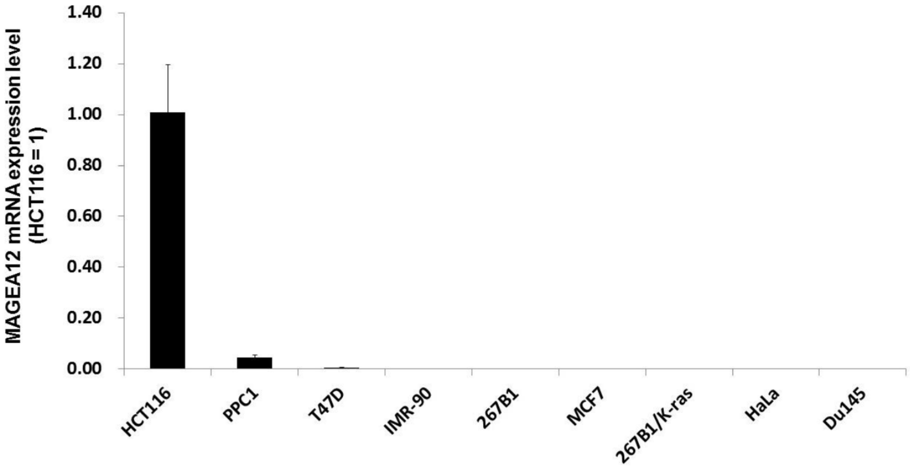

Supplementary Figure 3: MAGE-A12 expressed in colorectal cancer HCT116 and prostate cancer PPC1 cells. mRNA expression of MAGE-A12 in cancer cell lines was analyzed by qRT-PCR. mRNA levels of MAGE-A12 with normalization relative to GADPH are indicated (mean ± SD; n = 2).

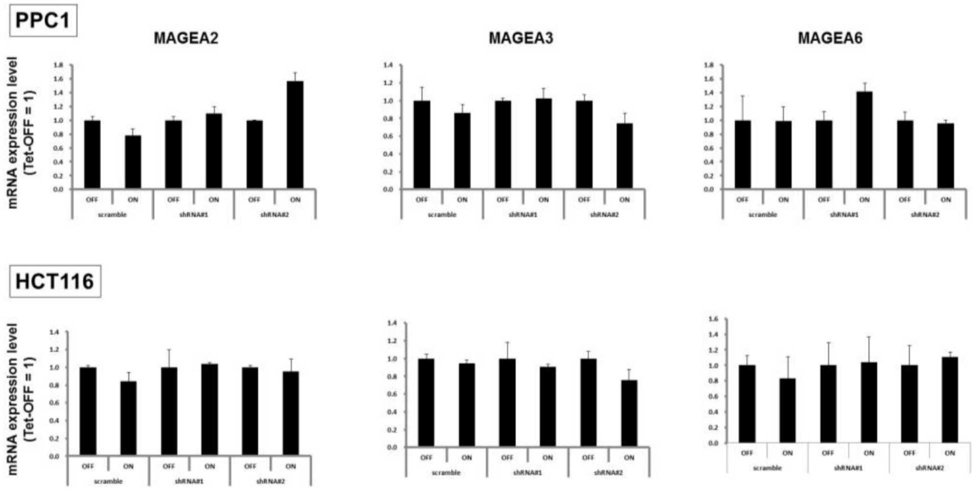

Supplementary Figure 4: shRNAs targeting MAGE-A12 do not affect the mRNA expression levels of other MAGE-A family genes. PPC1 and HCT116 cells stably containing inducible shRNAs targeting different sites on MAGE-A12 mRNA (shRNA#1, #2, and scramble control) were cultured for 2 days with (ON) or without (OFF) 100 ng/ml doxycycline (Dox). mRNA levels of MAGE-A2, -A3, and -A6 were measured by qRT-PCR, with normalization relative to GADPH (mean ± SD; n = 2).

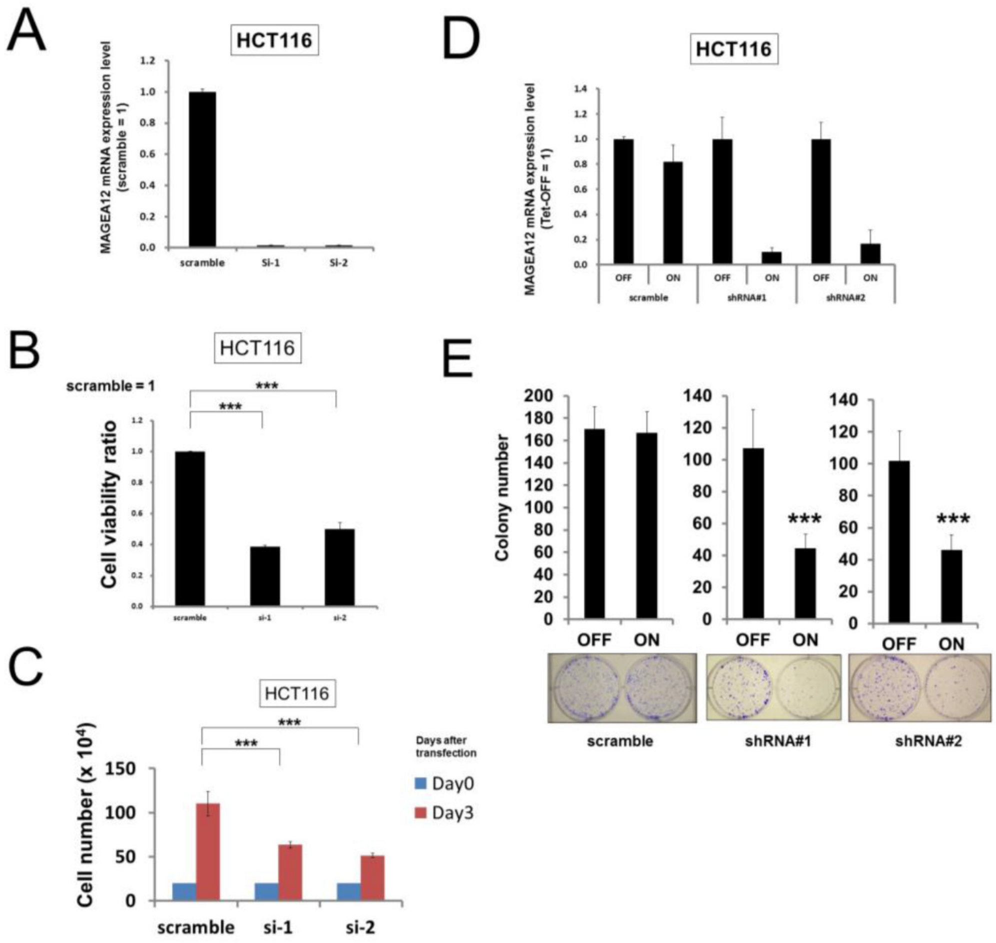

Supplementary Figure 5: Knockdown of MAGE-A12 mitigates the growth of colorectal cancer HCT116 cells. **(A)** Colorectal cancer HCT116 cells transfected with scrambled RNA or two different siRNAs targeting MAGE-A12 (siRNAs#1, #2). At 48 hours, the relative levels of MAGE-A12 mRNA were measured by qRT-PCR analysis. **(B)** HCT116 cells were transfected with control RNA or various siRNAs targeting MAGE-A12. At 72 hours, cellular ATP levels were measured as a surrogate indicator of relative number of viable cells, with data expressed as the ratio of values for cells transfected with MAGE-A12 to values for the control siRNAs (mean ± SD; n = 3). *** $p < 0.001$ by t-test. **(C)** To measure cell growth, 2.0 x 10^5 cells transfected with the indicated siRNAs were seeded onto 60-mm-diameter plates. At 72 hours, the numbers of cells were counted. *** $p < 0.001$ by t-test. **(D)** HCT116 cells stably containing inducible shRNAs targeting different sites on MAGE-A12 mRNA (shRNA#1, #2) were cultured for 48 hours with doxycycline (Dox: 100 ng/ml). Levels of MAGE-A12 mRNA were measured by qRT-PCR, with normalization relative to GADPH (mean ± SD; n = 2). **(E)** HCT116 cells stably containing inducible shRNAs were seeded at 300 cells per well in 60-mm dishes. After 24 hours, doxycycline (ON: 100 ng/ml, OFF: 0 ng/ml) was added. Colonies consisting of > 50 cells were enumerated on day 10. All data represent mean ± SD (n = 3). *** $p < 0.001$ by t-test.

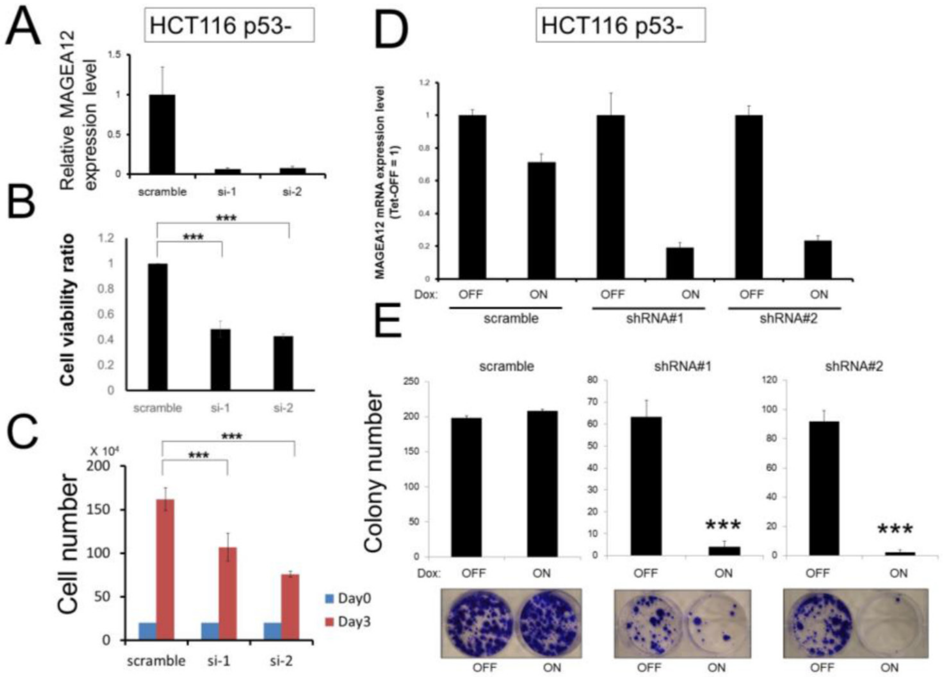

Supplementary Figure 6: Knockdown of MAGE-A12 mitigates the growth of p53-depleted HCT116 cells. **(A)** p53-depleted HCT116 (HCT116 p53-) cells transfected with scrambled RNA or two different siRNAs targeting MAGE-A12 (siRNAs#1, #2). At 48 hours, the relative levels of MAGE-A12 mRNA were measured by qRT-PCR analysis. **(B)** HCT116 p53- cells were transfected with control RNA or various siRNAs targeting MAGE-A12. At 72 hours, cellular ATP levels were measured as a surrogate indicator of relative number of viable cells, with data expressed as the ratio of values for cells transfected with MAGE-A12 to values for the control siRNAs (mean ± SD; n = 3). *** $p < 0.001$ by t-test. **(C)** To measure cell growth, 2.0×10^5 cells transfected with the indicated siRNAs were seeded onto 60-mm-diameter plates. At 72 hours, the cells were counted. *** $p < 0.001$ by t-test. **(D)** HCT116 p53- cells stably containing inducible shRNAs targeting different sites on MAGE-A12 mRNA (shRNA#1, #2) were cultured for 48 hours with doxycycline (Dox: 100 ng/ml). Levels of MAGE-A12 mRNA were measured by qRT-PCR, with normalization relative to GADPH (mean ± SD; n = 2). **(E)** HCT116 p53- cells stably containing inducible shRNAs were seeded at 300 cells per well in 60-mm dishes. At 24 hours, doxycycline (ON: 100 ng/ml, OFF: 0 ng/ml) was added. Colonies consisting of > 50 cells were enumerated on day 10. All data represent mean ± SD (n = 3). *** $p < 0.001$ by t-test.

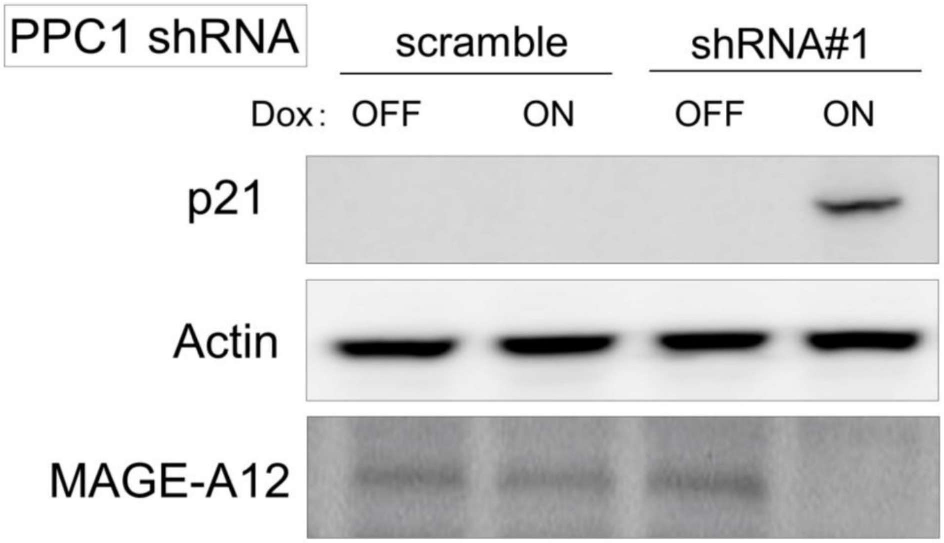

Supplementary Figure 7: Knockdown of MAGE-A12 induces the accumulation of p21. Protein lysates were generated from PPC1 cells (shRNA#1 and scramble control) cultured for 48 hours with or without 100 ng/ml doxycycline, normalized for total protein concentration, and analyzed by SDS-PAGE/immunoblotting using the indicated antibodies.

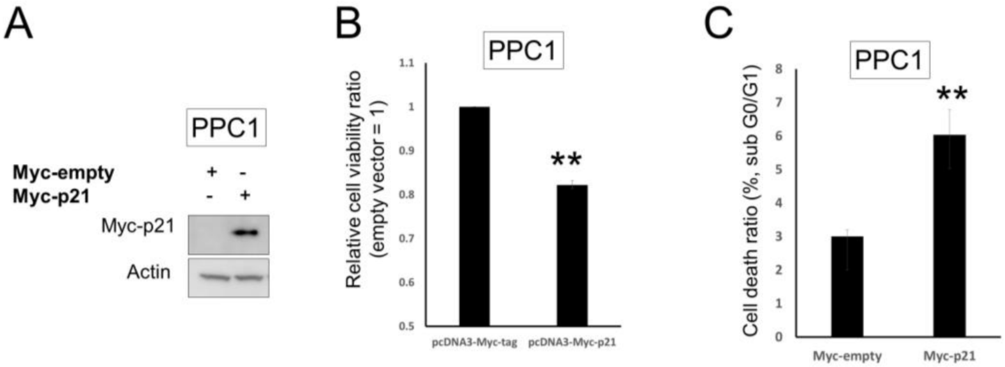

Supplementary Figure 8: Exogenous expression of p21 inhibits cell proliferation and induces cell death. (A) PPC1 cells were transfected with pcDNA3-Myc-p21 vector or control empty vector. At 48 hours, the cells were lysed in RIPA buffer, and the cell lysates were analyzed by immunoblotting. **(B)** Cellular ATP levels were measured as a surrogate indicator of the relative number of viable cells, with data expressed as the ratio of values for cells transfected with p21 to values for the control vectors (mean ± SD; n = 3). ** $p < 0.01$ by t-test. **(C)** FACS analysis was performed to assess the percentage of cell death. The % of sub G0/G1 is shown (mean ± SD; n = 3, ** $p < 0.01$).

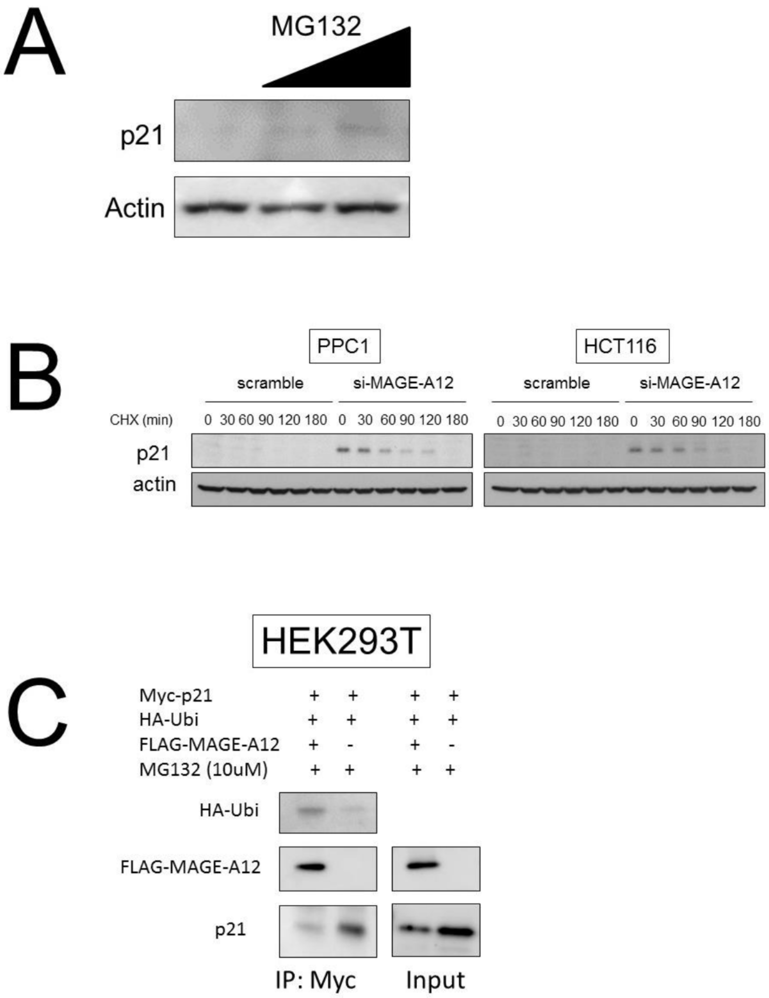

Supplementary Figure 9: MAGE-A12 regulates the ubiquitination of p21. **(A)** PPC1 cells were treated with or without MG132 (0, 5, 50 μM), and the cell lysates were harvested in RIPA buffer. An equal amount of protein from each lysate was analyzed by immunoblotting using rabbit anti-p21 antibody. **(B)** (Top) PPC1 and HCT116 cells were reverse-transfected with scramble or siRNA targeting MAGE-A12 (si-1). At 48 hours, cells were treated with 25 μg/ml cyclohexamide (CHX), and the cell lysates were harvested at the indicated times. **(C)** HEK293T cells were transfected with the indicated plasmids. At 48 hours, MG132 was added to cell culture (10 uM, 4 hours). The cells were lysed in buffer containing 1% NP-40, the cell lysates were subjected to immunoprecipitation (IP) with antibodies to Myc, and the resulting precipitates were subjected to immunoblot analysis with anti-HA or FLAG antibody. A portion (5%) of the lysates ("input") was also subjected directly to immunoblot analysis.
